# Effectiveness of person- and family-centered care transition interventions: a systematic review protocol

**DOI:** 10.1186/s13643-017-0554-z

**Published:** 2017-08-08

**Authors:** Chantal Backman, Julie Chartrand, Orvie Dingwall, Beverley Shea

**Affiliations:** 1 School of Nursing, Faculty of Health Sciences, University of Ottawa, 451, Smyth Rd, Ottawa, ON K1H 8M5 Canada; 2 Ottawa Hospital Research Institute, Ottawa, Canada; 3 University of Manitoba, Winnipeg, Canada; 4 Bruyere Research Institute, Ottawa, Canada

**Keywords:** Person- and family-centered care, Care transitions, Systematic review protocol

## Abstract

**Background:**

Care transitions from the hospital to home are critical to the sustainability of our health care system. Ineffective care transitions can be caused by high incidences of post-discharge adverse events, by poor communication with patients, and/or by inadequate information transfer between providers from the hospital to home. Any one of these can lead to fragmented care, high readmission rates, increased visits to the emergency department, and ultimately poor patient outcomes. Despite the ongoing improvement efforts of health care organizations, the efficacy of person- and family-centered care transition interventions on the quality of care and on the patient experience are not known. The aim of this systematic review is to critically analyze the body of evidence regarding the effectiveness of person- and family-centered care transition interventions on the quality of care, and the experience of patients.

**Methods:**

We will conduct a systematic review using the Cochrane Handbook’s guidelines and will adhere to a standardized reporting format: Preferred Reporting Items for Systematic Reviews and Meta-Analyses (PRISMA). A comprehensive search strategy will be conducted in the following databases: MEDLINE, EMBASE, CINAHL, Cochrane Central Register of Controlled Trials, and the Cochrane Consumers and Communication Group. Following a two-step screening process, data including the full reference, objectives, target population, description of the intervention and control intervention, outcome measures, design, length of post-intervention follow-up period, and the study results will be extracted, synthesized, and reported. Risk of bias and quality of the studies will also be assessed.

**Discussion:**

This systematic review will summarize and present the evidence base for person- and family-centered care transition interventions. This review will also inform further research and will lay the groundwork for more empirical studies on person- and family-centered care transitions. Specifically, the results of this systematic review may inform the development of measures to monitor safe and effective person- and family-centered transitions from the hospital to home. These results may also be important for policy makers, decision-makers, clinicians, and patients/families who are involved in navigating the health care system.

**Systematic review registration:**

PROSPERO CRD42017067990

**Electronic supplementary material:**

The online version of this article (doi:10.1186/s13643-017-0554-z) contains supplementary material, which is available to authorized users.

## Background

Care transitions from the hospital to home are critical to the sustainability of our health care system. Inadequate care transitions from the hospital to home are not uncommon as indicated by research demonstrating a high incidence of adverse events after hospitalization, as well as poor communication with patients and families during transitions and inadequate information exchange among health care providers [1–3]. All of these can lead to fragmented care, higher readmission rates, increased visits to the emergency department, and ultimately poor patient outcomes. Despite multiple person- and family-centered care interventions currently in place in hospitals, care transitions, particularly from the hospital to home, continue to be fragmented and to pose high safety risks [4].

Person- and family-centered care (PFCC) is defined as care that is “respectful of and responsive to individual patient preferences, needs, and values, and ensures that patient values guide all clinical decisions” [5]. Sidani and Fox have grouped PFCC processes into three components, which consist of holistic care, collaborative care, and responsive care [6]. Research shows that patients who are more involved in the decision-making process related to their care are better able to manage complex chronic conditions [7–9], have reduced anxiety and stress [10], and have shorter lengths of stay in the hospital [11]. Patient/family engagement is fundamental to a PFCC approach, and it is also key to improving overall patient care in our health care system [12]. Although there are several reviews that studied interventions for care transitions from the hospital to home [13–15], to date, only one review of the literature by Desai and colleagues [16] focused on examining the impact of PFCC transition processes from the hospital or the emergency department to the home. However, this paper exclusively looked at studies published in the USA, leading to a potential bias against countries with socialized medical care. Desai et al. [16] found four pediatric emergency department to home studies that demonstrated an association between tailored discharge education and patient outcomes and other studies that showed an association between a transition need assessment (*n* = 4) or an individualized transition care plan (*n* = 6) with better patient outcomes in the adult population [16]. To expand to countries with socialized medicine, and to conduct an expanded search of this previous literature review by adding further search terms (e.g., person- and family-centered care), we will critically analyze the body of evidence regarding the effectiveness of PFCC transition interventions from the hospital to home on the quality of care, and the experience of patients in the adult population. The specific objectives are:

1. To critically review the evidence on PFCC interventions on the quality of care, and experience of patients
2. To determine the effectiveness of PFCC interventions on improving the quality of care, and the experience of patients
3. To explore the effectiveness of these interventions on different population subgroups, if possible (e.g., male versus female participants), and/or different intervention types (e.g., patient versus family interventions).

## Methods

### Research design and methodology

We will conduct a systematic review (SR) to identify published articles regarding the impact of PFCC practices on the quality of care, and the experience of patients during care transitions between the hospital and home. This protocol has been prepared in accordance with the Preferred Reporting Items for Systematic review and Meta-Analysis Protocols (PRISMA-P) statement [17], as provided in Additional file 1. This SR is also registered with PROSPERO (registration # CRD42017067990).

### Eligibility criteria

To identify relevant studies, specific inclusion and exclusion criteria have been identified using the Population, Interventions, Comparators, Outcomes, and Study designs (PICOS) criteria as follows: *Population*: adult population (18 years of age or older). *Interventions*: any PFCC transition interventions from an inpatient hospital unit to home (e.g., individualized discharge plan, individualized transition record, post-discharge telephone follow-up, home visits, patient- and family-tailored discharge information, and transition need assessment) based on the components of the PFCC framework, which include holistic, collaborative, and responsive care [6]. PFCC transition interventions from emergency departments to home will be excluded. *Comparator*: usual care. *Outcome*: quality of care measures and patient-reported experience measures (PREMs) (i.e., discharge readiness, functional status, adverse events, health-related quality of life, knowledge of care plan, medication adherence, adherence to follow-up, 30-day emergency department visits, 30-day readmissions, satisfaction [16]). *Study designs*: randomized controlled trials and non-randomized experimental studies (e.g., cohort, case-control, controlled before-and-after, interrupted-time-series, and controlled trials not using full randomization).

### Search strategy

An experienced health sciences information specialist (OD) developed the preliminary MEDLINE and CINAHL search strategies utilizing search terms on person- and family-centered care and care transitions. The preliminary search strategies were conducted on November 25, 2016, and identified 6130 records. The MEDLINE search is included in the Appendix. During our preliminary review, we identified, for the period of “2015 to current,” 23/358 (6%) records that were relevant, confirming the feasibility of this SR. The search strategy was peer reviewed by an external experienced librarian using the Peer Review of Electronic Search Strategies checklist [18]. After this exercise, the literature search was modified as necessary. Databases to be searched will include, but are not limited to, the following: MEDLINE (1946–current), EMBASE (1980–current), CINAHL (1982–current), Cochrane Central Register of Controlled Trials, and the Cochrane Consumers and Communication Group. Supplementary searches of key journal and of gray literature websites will be undertaken. All results will be imported into a citation management software, where duplicate citations will be screened and removed.

### Screening

We will use a two-step process to assess the results of the literature search. We will develop screening questions based on the inclusion/exclusion criteria for both screening levels. Prior to conducting the formal screening, a calibration exercise will be undertaken to pilot test and refine our screening questions. Screening will be done using Covidence systematic review software [19]. A level 1 screening will be performed independently with two reviewers screening records according to the pre-specified criteria. All marginally relevant records and those records that do not contain enough information to determine eligibility (e.g., no available abstract) will be retained. Conflicts will be resolved by consensus or by a third reviewer. For the level 2 screening, two reviewers will independently assess the full text of all retained records. Discrepancies will be resolved by consensus or by a third reviewer. The reasons for exclusion will be noted using the Preferred Reporting Items for Systematic Reviews and Meta-Analyses (PRISMA) format [20].

### Data extraction

Prior to performing data extraction, a calibration exercise will be undertaken to pilot test and refine our data extraction form. Two reviewers will independently extract and document data from each included study using a standardized data extraction form in the Covidence systematic review software. We will extract data including the full reference, objectives, target population, description of the intervention and control intervention, outcome measures, design, length of post-intervention follow-up period, and study results.

### Assessing risk of bias

Two reviewers will independently assess the risk of bias (RoB) associated with each included study. We will appraise the risk of bias using two validated tools depending on the study design. For randomized trials, we will assess RoB using the Cochrane RoB tool. This tool evaluates seven domains (i.e., sequence generation, allocation concealment, blinding of participants and personnel, blinding of outcome assessment, missing outcome data, selective outcome reporting, and other sources of bias) [21]. Studies will be evaluated as “low” (unlikely plausible risk of bias that could alter confidence in the results), “unclear” (plausible bias that raise a doubt of the validity of the results), or “high” (plausible bias that seriously weakens the confidence in the results) as per the criteria. For non-randomized experimental studies (e.g., cohort, case-control, controlled before-and-after, interrupted-time-series, and controlled trials not using full randomization), we will use a recently developed Risk of Bias In Non-Randomized Studies of Interventions (ROBINS-I) tool [22]. It consists of seven domains (i.e., confounding, participant selection, intervention measurement, departures from intended interventions, missing data, outcome measurement, and selection of reported results). Studies will be evaluated as low (low in all domains), moderate (low to moderate in all domains), serious (serious in at least one domain, but not critical), critical (critical in at least one domain), and no information. Studies will be assessed for selection, performance, detection, attrition, reporting, and other biases. Disagreements will be resolved through discussion and, if necessary, by consulting a third reviewer.

### Assessing the quality of the evidence

We will use the Grading of Recommendations Assessment, Development and Evaluation (GRADE) framework to assess the quality of the body of evidence [23]. Specifically, the overall certainty in the evidence, which is based on our confidence that the estimates of the effect are correct, will be assessed for each outcome identified across studies using the four categories (high, moderate, low, and very low) [23].

### Analysis/synthesis

We will tabulate and discuss data in a narrative review. We will organize data by categories, grouping studies by settings, disease type, intervention type, outcome, and study design [24]. All data tables will contain data on setting, intervention and control, study sample, patient characteristics, disease, study design, outcome, and overall RoB. Where appropriate, meta-analysis of results from randomized controlled studies and non-randomized studies will be carried out to estimate a summary measure of effect. *Statistical analysis*: For each study, we will calculate risk ratios and the 95% confidence interval for dichotomous outcomes. Mean differences for continuous variables and 95% confidence intervals will be calculated for continuous outcomes. If the scale for each assessment varies among the studies, we will calculate a standardized mean difference based on end-of-study results. Heterogeneity between comparable studies will be tested using a standard chi^2^ test and considered statistically significant at *P* < 0.05; after due consideration of the value of the *I* ^2^ statistic, a value greater than 50% may indicate substantial heterogeneity. *Data synthesis*: Where appropriate, results of comparable groups of studies will be pooled using the fixed-effect model and 95% confidence intervals will be calculated. If heterogeneity exists between studies, a random-effect model will be used. Meta-analysis will be performed using Review Manager 5 [25]. *Subgroup and sensitivity analyses*: If sufficient data is available from the included studies, subgroup analyses will be conducted to compare the effect of interventions on different population subgroups (e.g., male versus female participants) and/or different intervention types (e.g., patient versus family interventions). Where statistical pooling is not possible, the findings will be presented in narrative form. However, we are unable to specify the subgroups in advance.

### Quality assurance

The proposed SR will be reported using the PRISMA format [20]. PRISMA consists of a checklist of 27 essential items for transparent reporting of SRs [20]. In addition, we will use the AMSTAR 2, 16-item checklist (developed as a critical appraisal tool for SRs) as a guide to ensure our SR meets quality standards and to avert any possible deficiencies in the conduct and reporting of our review [26].

### Potential challenges and mitigation strategies

SRs are designed to follow a meticulous, clearly defined methodology that is explicit in all its analytical steps. It is these features that make a SR one of the most robust ways of producing reliable evidence. In conducting such a review, it is important to consider how to mitigate challenges such as the following: *missing data*: Where the outcome data is unclear, incompletely reported, or not reported, we will attempt to contact the study authors to obtain the data. The extent of Missing data will be documented in our RoB tables. *Articles published in other languages*: For articles not published in English, we will attempt to find translators or English translations. For French articles, CB and JC will review the abstracts to determine inclusion eligibility.

## Discussion

This systematic review will summarize and present the evidence base for person- and family-centered care transition interventions from the hospital to home in the adult population. Although a previous review [16] has found some intervention studies on this topic, that review was solely focused on the studies from the USA. This planned review will serve to identify additional person- and family-centered interventions and expand the scope of the SR to include more recent international studies, to gain a perspective from socialized medical systems. This review will also inform further research and will lay the groundwork for more empirical studies on person- and family-centered care transitions. Specifically, the results of this SR may inform the development of measures to monitor safe and effective PFCC transitions from the hospital to home. These results may also be important for policy makers, decision-makers, clinicians, and patients/families who are involved in navigating the health care system.

## Appendix

### Preliminary MEDLINE search strategy

1. exp “continuity of patient care”/
2. (continu* adj3 care).ti,ab,kw,kf,hw.
3. discharge*.ti,ab,kw,kf,hw.
4. handoff*.ti,ab,kw,kf,hw.
5. hand off*.ti,ab,kw,kf,hw.
6. handover*.ti,ab,kw,kf,hw.
7. hand over*.ti,ab,kw,kf,hw.
8. signoff*.ti,ab,kw,kf,hw.
9. sign off*.ti,ab,kw,kf,hw.
10. signover*.ti,ab,kw,kf,hw.
11. sign over*.ti,ab,kw,kf,hw.
12. transfer*.ti,ab,kw,kf,hw.
13. transition*.ti,ab,kw,kf,hw.
14. (turf* adj3 patient*).ti,ab,kw,kf,hw.
15. (dump* adj3 patient*).ti,ab,kw,kf,hw.
16. posthospital*.ti,ab,kw,kf,hw.
17. post hospital*.ti,ab,kw,kf,hw.
18. or/1-17
19. home.ti,ab,kw,kf,hw.
20. exp home care services/
21. (domicil* adj3 care).ti,ab,kw,kf,hw.
22. patient readmission/
23. readmi*.ti,ab,kw,kf,hw.
24. rehospitali*.ti,ab,kw,kf,hw.
25. post discharge.ti,ab,kw,kf,hw.
26. or/19-25
27. exp patient-centered care/
28. (patient* adj3 (centred* or centered* or focus*)).ti,ab,kw,kf,hw.
29. (person adj3 (centred* or centered* or focus*)).ti,ab,kw,kf,hw.
30. (famil* adj3 (centred* or centered* or focus*)).ti,ab,kw,kf,hw.
31. patient participation/
32. (patient* adj3 (involve* or empower* or participat* or activat* or engage* or perspective*)).ti,ab,kw,kf,hw.
33. patient education as topic/
34. (patient* adj3 educat*).ti,ab,kw,kf,hw.
35. quality improvement/
36. quality improvement.ti,ab,kw,kf,hw.
37. (intervent* adj3 (care or patient*)).ti,ab,kw,kf,hw.
38. or/27-37
39. 18 and 26 and 38
40. remove duplicates from 39

## Additional file

Additional file 1: PRISMA-P 2015 checklist. (DOCX 39 kb)

## Abbreviations

GRADE: Grading of Recommendations Assessment, Development and Evaluation
PFCC: Person- and family-centered care
PICOS: Population, Interventions, Comparators, Outcomes, and Study designs
PREMs: Patient-reported experience measures
PRISMA: Preferred Reporting Items for Systematic Reviews and Meta-Analyses
PRISMA-P: Preferred Reporting Items for Systematic review and Meta-Analysis Protocols
RoB: Risk of bias
ROBINS-I: Risk of Bias In Non-Randomized Studies of Interventions
SR: Systematic review
